# PAM50 breast cancer subtypes and survival of patients in rural Ethiopia without adjuvant treatment: a prospective observational study

**DOI:** 10.1186/s12885-024-12867-6

**Published:** 2024-09-10

**Authors:** Judith Katharina Ballé, Martina Vetter, Tariku Wakuma Kenea, Pia Eber-Schulz, Christian Reibold, Hannes-Viktor Ziegenhorn, Kathrin Stückrath, Claudia Wickenhauser, Adamu Addissie, Pablo Santos, Eva Johanna Kantelhardt, Sefonias Getachew, Marcus Bauer

**Affiliations:** 1 Global Health Working Group, Institute of Medical Epidemiology, Biostatistics and Informatics, Martin Luther University Halle-Wittenberg, Halle (Saale), Germany; 2 Department of Gynaecology, Martin Luther University Halle-Wittenberg, Halle (Saale), Germany; 3 Department of Surgery, Aira General Hospital, Aira, Ethiopia; 4 Department of Anesthesiology and Critical Care Medicine, Carl Gustav Carus University Hospital, Dresden, Germany; 5 Institute of Pathology, Martin Luther University Halle-Wittenberg, Halle (Saale), Germany; 6 Department of Epidemiology and Biostatistics, School of Public Health, College of Health Sciences, Addis Ababa University, Addis Ababa, Ethiopia

**Keywords:** Africa, Ethiopia, Breast Cancer, PAM50, Intrinsic subtypes, Survival

## Abstract

**Purpose:**

Survival rates of breast cancer (BC) patients are particularly low in rural regions in sub-Saharan Africa (SSA) which is due to limited access to therapy. In recent years, gene expression profiling (GEP) of BC showed a strong prognostic value in patients with local tumour surgery and (neo)adjuvant treatment. The aim of this study was to evaluate the impact of intrinsic subtypes on survival of patients in rural Ethiopia without any (neo)adjuvant therapy.

**Methods:**

In total, 113 female patients from Aira Hospital with histologically proven BC and treated only with surgery were included in this study. All samples were analysed by immunohistochemistry (IHC) for estrogen receptor, progesterone receptor, HER2 and Ki67, as well as RNA-expression analysis for PAM50 subtyping.

**Results:**

A positive hormone receptor status was found in 69.0% of the tumours and intrinsic subtyping demonstrated Luminal B to be the most common subtype (34.5%). Follow-up data was available for 79 of 113 patients. Two-year overall survival (OS) was 57.3% and a considerably worse OS was observed in patients with Basal-like BC compared to Luminal A BC. Moreover, advanced tumours showed an increased risk of mortality.

**Conclusion:**

The OS was very low in the patient cohort that received no (neo)adjuvant treatment. Immunohistochemistry and GEP confirmed endocrine-sensitive tumours in more than half of the patients, with a large proportion of Luminal B, HER2-enriched and Basal-like tumours so that adjuvant chemotherapy should be recommended.

**Supplementary Information:**

The online version contains supplementary material available at 10.1186/s12885-024-12867-6.

## Introduction

Breast cancer (BC) is the most common malignancy among women in sub-Saharan Africa (SSA) and the second leading cause of cancer-related deaths [1]. In comparison to high income countries, survival of BC patients is much lower for most countries in SSA [2, 3]. Previous studies from Ethiopia showed two-year survival numbers between 53% and 74% and a 5-year survival of 46% [4]. In contrast, countries with more developed health care systems observe survival rates of around 90% after 5 years [5]. Various factors contribute to these disparities, amongst them late stages of presentation as well as limited diagnostic and treatment capabilities [6, 7].

Gene expression profiling (GEP) has had a major impact on gaining a better understanding of the heterogeneity of breast cancer. GEP may help to identify distinct molecular signatures that demonstrated prognostic impact for BC patients [8]. Later, Parker and colleagues simplified the profiling algorithms using 50 genes for classification of the intrinsic subtypes [9]. Nowadays, four subtypes, namely Luminal A, Luminal B, Human Epidermal Growth Factor 2 (HER2)-enriched and Basal-like subtype can be distinguished, which have been studied in systemically treated patients [8]. By employing GEP, significant disparities in the proportion of the subtypes in different populations have been reported in recent years. This was associated with varied risk factors and therapy sensitivity as well as risk of recurrence and mortality [10]. Insights gained through GEP can add valuable information about prognosis and presumable response to therapy. For instance, patients with Luminal A subtype show a high sensitivity for endocrine treatment, meanwhile response to hormone therapy only is much lower among patients with Luminal B subtype, who additionally need chemotherapy to improve their prognosis [11].

However, GEP based BC subtyping is expensive and has so far not been implemented in most low- and middle-income countries (LMICs). As a surrogate, histopathology and immunohistochemistry (IHC) for estrogen receptor (ER), progesterone receptor (PgR), HER2, and Ki67 proliferation index is commonly used to describe the different BC groups and provides prognostic information to facilitate treatment decision making. Numerous studies have investigated the prevalence of IHC-based BC groups in SSA, pointing out regional differences of hormone receptor expression of BC [12]. Nevertheless, it could be demonstrated that grouping based on IHC is inferior in providing prognostic information compared to gene expression subtyping, which could lead to wrong treatment decisions and poorer survival rates [13–17]. However, IHC is not readily available in SSA [7]. In Ethiopia, the availability of IHC is limited to a few central hospitals and this limitation is more severe in rural areas [18]. Therefore, data is needed to gain a better insight into the tumour biology and consequently to improve breast cancer survival through a more personalised therapy in SSA.

The aim of this study was to analyse whether IHC grouping and intrinsic subtyping predicts patients’ survival in a cohort of BC patients without (neo)adjuvant treatment. Our prior work found a large proportion of tumours to be hormone-receptor positive [19]. We therefore assumed that GEP determines numerous Luminal subtypes. For that reason, we utilised PAM50 gene expression assay for intrinsic breast cancer subtyping and evaluated the prevalence of intrinsic subtypes in comparison to IHC grouping and further histopathological factors like grading and their impact on overall survival (OS) in a cohort of BC patients without any (neo)adjuvant therapy.

## Methods

### Study design, patients, and tumour characteristics

**Fig. 1:**
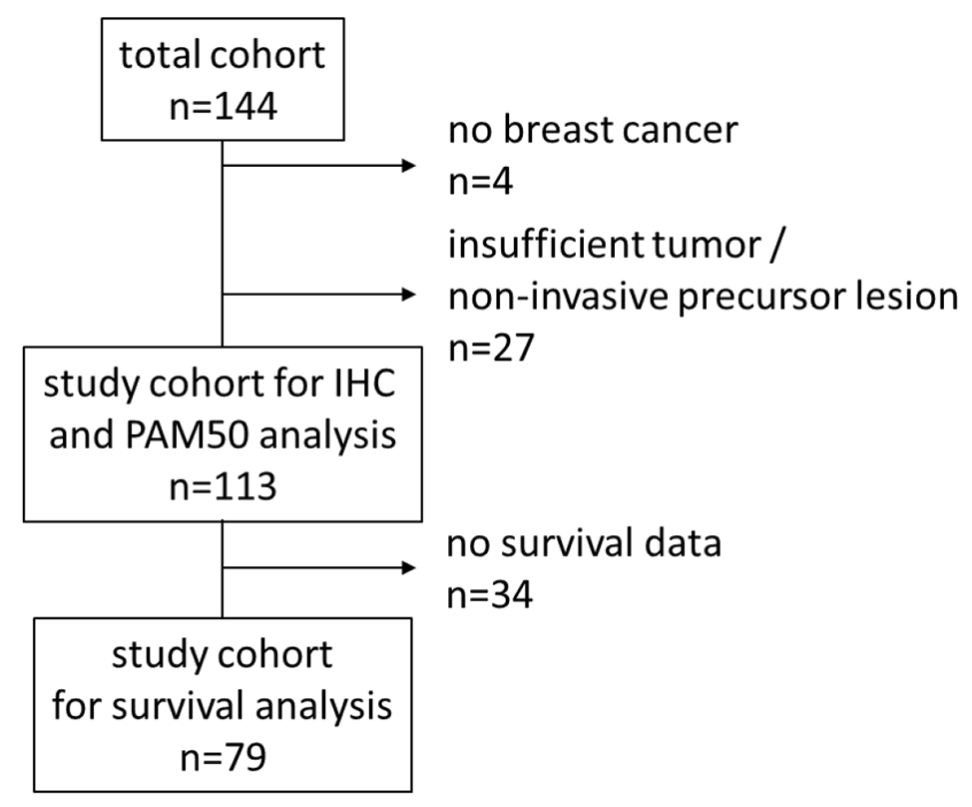
Enrolment of the study cohort

Female BC patients were included in this study in accordance with the REMARK criteria [20]. Tissue specimens were prospectively collected from 144 patients at the Aira General Hospital, Ethiopia, between 2010 and 2018. Aira Hospital is a primary level hospital located approximately 500 km west of the capital Addis Ababa in the rural Oromyia region. The facility serves approximately 500,000 people and performs approximately 90 breast surgeries annually. The only available treatment option during the time was surgery. Adjuvant chemotherapy and radiation were not available in Aira. All patients had clinically suspicious breast tumour lesions and underwent surgery without prior core needle biopsy. Of these 144 patients, 31 were excluded due to benign BC tumour lesions (*n* = 4), insufficient tumour tissue or non-invasive precursor lesions (*n* = 27). For the cross-sectional analysis, 113 BC patients were identified (Fig. 1). Patients’ demographic and clinicopathologic data was collected from medical records. Clinical and histopathological data are summarized in Table 1. Follow-up data were available for 79 patients, with a median follow-up time of 22 months (range 1 day – 72.3 months).

### Histopathology and immunohistochemistry (IHC)

Formalin-fixed and paraffin embedded (FFPE) specimens were centrally analysed at the Institute of Pathology, University Hospital Halle, Martin Luther University Halle-Wittenberg, Germany. All samples were analysed by histomorphology using haematoxylin and eosin (H&E) staining for histological diagnosis according to the World Health Organization (WHO) classification of breast tumours, 5th Edition, 2019. Grading was determined according to Nottingham grading system by Elston and Ellis [21]. All samples were analysed by conventional IHC using antibodies directed against the estrogen receptor (ER), progesterone receptor (PgR), HER2 and Ki67 (Supplementary Table S1). IHC staining was performed on a Bond III automated immunostainer (Leica Biosystems Nussloch GmbH, Wetzlar, Germany) using the Bond Polymer Refine Detection Kit (DS9800-CN). Expression analysis of ER and PgR status was performed according to the current guidelines [22]. A negative ER or PgR status was declared as receptor expression of < 1% of tumour cells. If at least one of the markers was positive, the hormone receptor status was defined as positive. HER2 status was assessed according to the American Society of Clinical Oncology and College of American Pathologists (ASC-CAP) guidelines [23]. The Ki67 proliferation index was visually classified as high if more than 25% of the tumour cells stained positive [24]. BC subtypes were classified according to the surrogate IHC group classification using hormone receptor expression of ER and PgR, the HER2 status as well as Ki67 proliferation index including the following IHC groups: Luminal A-like, Luminal B-like, HER2 positive, and triple negative BC (TNBC) [25].

### RNA isolation and expression analysis

Prior to RNA isolation, FFPE specimens were microdissected. For RNA isolation, two to four 10-µm thick tissue slides were used. Deparaffinisation of FFPE tissues was performed 2 times with xylene for 5 min, followed by incubation in 96% and 70% ethanol for 2 min each. Proteinase K digestion was performed for up to 2 h at 56 °C followed by 15 min at 80 °C. RNA was isolated with miRNeasy Mini FFPE Kit^®^ (Qiagen, Venlo, The Netherlands) according to the manufacturer’s instructions. RNA expression analysis was performed using the NanoString Assay according to the hybridisation protocol for the nCounter^®^ XT CodeSet Gene Expression Assay (NanoString nCounter^®^, Seattle, WA, USA). Following analysis of data was performed according to the nCounter Expression Data Analysis Guide (MAN- C0011-04 from 2017). The expression levels were appraised using the R package NanoStringNorm (github.com/sgrote/NanoStringNormalizeR/). The PAM50 gene algorithm was used to determine the intrinsic breast cancer subtypes [9].

### Endpoints and statistical analysis

The study’s first objective was to evaluate the distribution of intrinsic subtypes in BC in a cohort of rural Ethiopian patients. The second objective was to evaluate the impact of intrinsic subtypes to clinical outcome with regard to OS of patients without any (neo)adjuvant treatment. OS included deaths from breast cancer, non-breast cancer, and unknown causes [26]. If survival status was not clear, patients were censored at the last timepoint of contact. OS analysis was performed using the Kaplan-Meier estimator method and the log-rank test. The prognostic value was determined by univariate and multivariate Cox’s proportional hazards models. Clinically relevant prognostic factors included in the model were age, Nottingham grade, tumour size, lymph node status and PAM50 subtypes. The hazard ratios (HR) were presented with 95% confidence intervals (CI), p values below 0.05 were considered statistically significant. Statistical analyses were performed using SPSS 28 (IBM, Armonk NY, USA).

## Results

### General epidemiological and clinical characteristics

A total of 113 patients from rural Ethiopian regions were included in this study. The median age of patients at time of diagnosis was 42 years (ranging between 16 and 80). Around 75% of patients reported being illiterate and four out of five women lived in rural areas outside the town of Aira. The mean walking time to reach the local hospital was 6.5 h. Only 11.5% of women lived in the urbanised Aira region, 55.7% lived in a rural area and of 11.5% of patients the site of residence was unknown. The mean number of births per woman was 4.8 and 90% of them reported breastfeeding (with a mean total duration of 11.5 years or 2.4 years breastfeeding per child). Nearly half of patients reported having had symptoms for more than 12 months before first consulting a doctor.

Clinical information was available of 76 patients (67.2%), and most patients (*n* = 44; 57.9%) presented clinically at advanced stage with tumour size larger than 5 cm in diameter. Clinical lymph node involvement was observed in 80.5% of cases. Distant metastases were clinically reported for six patients. No diagnostic imaging or pathological analysis were available locally. No radiotherapy and no chemotherapy were reported. Detailed information is given in Table 1.

**Table 1:** Epidemiological and clinical characteristics of the cohort

Characteristics (*n* with information available)	*n* (% or range)
age at diagnosis	42 years (range: 16–80)
<35	30 (26.5%)
35–50	61 (53.9%)
> 50	17 (15.1%)
missing data	17 (15.1%)
time of diagnosis	
2009–2011	38 (33.6%)
2012–2014	40 (35.4%)
2015–2018	35 (31.0%)
site of residence	
rural	63 (55.7%)
town	13 (11.5%)
missing data	37 (32.8%)
number of births (*n* = 64)	4.8 (range: 0–12)
number of children (*n* = 79)	4.1 (range: 0–11)
time to presentation	
≤ 12 months	38 (33.6%)
> 12 months	31 (27.4%)
missing data	44 (39.0%)
clinical tumour size	
cT1 (< 2 cm)	4 (3.5%)
cT2 (≥ 2 cm, < 5 cm)	29 (25.7%)
cT3 (≥ 5 cm)	29 (25.7%)
cT4	15 (13.3%)
missing data	36 (31.8%)
clinical lymph node status	
cN negative	22 (19.5%)
cN positive	91 (80.5%)
distant metastases	
cM negative	73 (64.6%)
cM positive	6 (5.3%)
missing data	34 (30.1%)
surgical treatment	
simple mastectomy	9 (8.0%)
modified radical mastectomy	22 (19.5%)
radical mastectomy	3 (2.7%)
lumpectomy	8 (7.1%)
quadrantectomy	8 (7.1%)
mastectomy (not specified)	18 (15.9%)
missing data	45 (39.8%)
stage*	
localized	20 (17.7%)
local spread	1 (0.9%)
regional spread	49 (43.4%)
advanced	6 (5.3%)
missing data	37 (32.7%)
observation time (*n* = 79)	
median OS	22 months (1 day – 72.3 months)
mean OS	34 months (SD 18.1 months)

*According to European Network of Cancer Registries (ENCR) recommendations (condensed TNM for coding the extent of disease)

### Immunohistochemical analysis and molecular subtyping revealed a high prevalence of hormone receptor positive and Luminal breast cancer

**Fig. 2:**
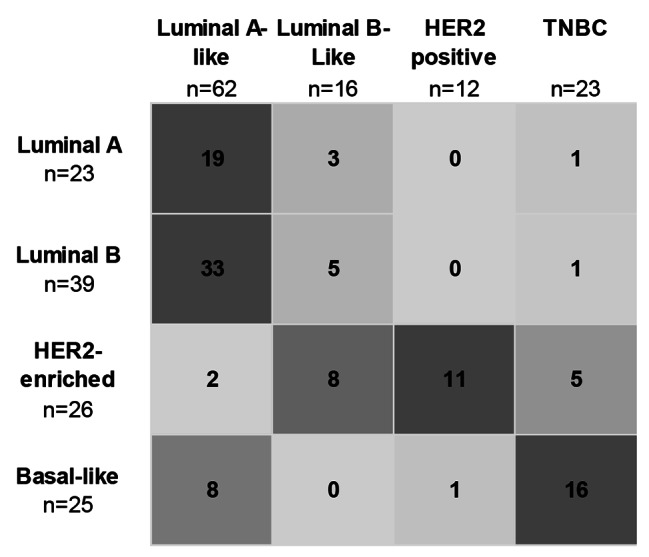
Comparison of IHC groups and intrinsic subtypes. Color-coded cross table of 113 BC tissue samples grouped according to IHC groups (columns) and intrinsic subtypes (rows). The cell colour gradient indicates the relationship in terms of the numbers of samples that fit in both classifications (IHC groups and intrinsic subtyping)

Histological assessment revealed a predominance of invasive BC of no special type (NST) which was identified in 109 patients (96.5%). The vast majority of patients showed a Nottingham grade 3 (72.6%). An invasion of lymphatic or blood vessels was observed in 66.4% and 8.9% of cases, respectively. IHC analysis for 113 BC specimens demonstrated that 58.4% and 54.9% of the tumours were ER and PgR positive, respectively. The prevalence of HER2 positive tumours was 24.8% and for TNBC was 20.4%. A high proliferation index of Ki67 staining (cut off > 25%) was observed in 61.9%. Next, molecular subtyping using the PAM50 gene expression algorithm revealed Luminal B subtype to be the most common subtype in this cohort with a frequency of 34.5%. HER2-enriched tumours were determined in 23.0% of samples. The prevalence of Basal-like and Luminal A subtype was 22.1% and 20.4%, respectively (Table 2). Comparison of IHC groups and intrinsic subtypes is shown in Fig. 2. This analysis highlights a low concordance between Luminal B-like and Luminal B classification. This low agreement is also highlighted by a Cohen’s kappa of 0.167 (*p* < 0.001).

**Table 2:** Histological, immunohistochemical characteristics and intrinsic subtype distribution if the cohort

Characteristics	*n* (%)
histological type	
NST	109 (96.5%)
other	4 (3.5%)*
Nottingham grade	
grade 1	2 (1.8%)
grade 2	29 (25.6%)
grade 3	82 (72.6%)
lymphatic vessel invasion	
no	38 (33.6%)
yes	75 (66.4%)
blood vessel invasion	
no	103 (91.1%)
yes	10 (8.9%)
hormone receptor status	
negative	35 (31.0%)
positive	78 (69.0%)
ER status	
negative	47 (41.6%)
positive	66 (58.4%)
PgR status	
negative	51 (45.1%)
positive	62 (54.9%)
HER2 status	
negative	85 (75.2%)
positive	28 (24.8%)
Ki67 proliferation index	
≤25%	43 (38.1%)
>25%	70 (61.9%)
IHC groups	
Luminal A-like	62 (54.9%)
Luminal B-like	16 (14.2%)
HER2 positive+	12 (10.6%)
TNBC	23 (20.3%)
PAM50 subtypes	
Luminal A	23 (20.4%)
Luminal B	39 (34.5%)
HER2-enriched	26 (23.0%)
Basal-like	25 (22.1%)

NST, no special type of carcinoma; ER, estrogen receptor; PgR, progesterone receptor; HER2, human epidermal growth factor receptor 2; * other histological diagnosis included invasive lobular carcinoma and mucinous carcinoma

No differential distributions were detected for age, site of residence, tumour size and stage in the four intrinsic subtypes (see Table 3). However, Basal-like tumours showed higher histopathological grading, more often negative hormone receptor status, and a higher Ki67 proliferation index compared to non-Basal-like tumours. Furthermore, a lower frequency of positive HER2 status was found in these tumours when compared with HER2-enriched subtype.

**Table 3:** Epidemiological, clinical, and pathological characteristics, stratified by intrinsic subtypes

Characteristics (*n*)	Luminal A *n* (%)	Luminal B *n* (%)	HER2-enriched *n* (%)	Basal-like *n* (%)	*p*-value (Fisher’s exact test)
age (years)					0.906
< 35 (30)	6 (27.3%)	13 (33.3%)	7 (28.0%)	4 (18.2%)	
35–50 (61)	13 (59.1%)	21 (53.8%)	13 (52.0%)	14 (63.6%)	
> 50 (17)	3 (13.6%)	5 (12.8%)	5 (20.0%)	4 (18.2%)	
site of residence					0.187
rural (63)	9 (69.2%)	26 (89.7%)	14 (73.7%)	14 (93.3%)	
urban (13)	4 (30.8%)	3 (10.3%)	5 (26.3%)	1 (6.7%)	
clinical tumour size					0.103
cT1, < 2 cm (4)	1 (7.1%)	3 (10.3%)	0	0	
cT2, ≥ 2 cm, < 5 cm (29)	8 (57.1%)	7 (24.1%)	9 (47.7%)	5 (33.3%)	
cT3, ≥ 5 cm (29)	4 (28.6%)	12 (41.4%)	9 (47.7%)	4 (26.7%)	
cT4 (15)	1 (7.1%)	7 (24.1%)	1 (5.3%)	6 (40.0%)	
lymph node status					0.454
cN negative (22)	6 (42.9%)	6 (20.7%)	5 (26.3%)	5 (33.3%)	
cN positive (55)	8 (57.1%)	23 (79.3%)	14 (73.7%)	10 (66.7%)	
distant metastases					0.785
cM negative (73)	14 (100.0%)	28 (90.3%)	17 (89.5%)	14 (93.3%)	
cM positive (6)	0	3 (9.7%)	2 (10.5%)	1 (6.7%)	
stage*					0.618
localized (20)	6 (42.9%)	4 (14.3%)	5 (26.3%)	5 (33.3%)	
local Spread (1)	0	1 (3.6%)	0	0	
regional Spread (49)	8 (57.1%)	20 (71.4%)	12 (63.2%)	9 (60.0%)	
advanced (6)	0	3 (10.7%)	2 (10.5%)	1 (6.7%)	
Nottingham grade					**< 0.001**
grade 1 (2)	2 (8.7%)	0	0	0	
grade 2 (29)	14 (60.9%)	9 (23.1%)	4 (15.4%)	2 (8.0%)	
grade 3 (82)	7 (30.4%)	30 (76.9%)	22 (84.6%)	23 (92.0%)	
hormone receptor status					**< 0.001**
negative (35)	1 (4.3%)	1 (2.6%)	16 (61.5%)	17 (68.0%)	
positive (78)	22 (95.7%)	38 (97.4%)	10 (38.5%)	8 (32.0%)	
HER2 Status					**< 0.001**
negative (85)	20 (87.0%)	34 (87.2%)	7 (26.9%)	24 (96.0%)	
positive (28)	3 (13.0%)	5 (12.8%)	19 (73.1%)	1 (4.0%)	
Ki67 proliferation index					**< 0.001**
≤25% (43)	19 (82.6%)	11 (28.2%)	6 (23.1%)	7 (28.0%)	
>25% (70)	4 (17.4%)	28 (71.8%)	20 (76.9%)	18 (72.0%)	
vital status					0.844
dead (42)	6 (42.9%)	17 (54.8%)	10 (52.6%)	9 (60.0%)	
alive (37)	8 (57.1%)	14 (45.2%)	9 (47.4%)	6 (40.0%)	

ER, estrogen receptor; PgR, progesterone receptor; HER2, human epidermal growth factor receptor. *According to ENCR recommendations (condensed TNM for coding the extent of disease)

### Survival analysis showed only minor differences with inferior survival in Basal-like subtype

**Fig. 3:**
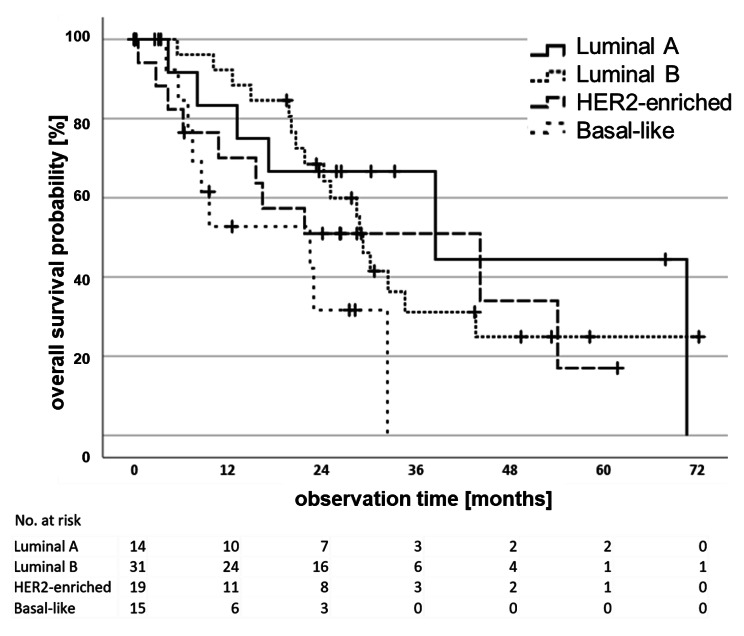
Overall survival analysis for 79 patients with regard to intrinsic subtypes

Follow-up data were available for 79 of 113 patients. The mean OS time was 34 months, and the median OS time was 22 months (ranging from 0 to 72). After two years, 57.3% were still alive (95% confidence interval 45.3-69.3%). The subtype-specific mean OS of the patients with Luminal A, Luminal B, HER2-enriched and Basal-like tumours was 43.6, 36.7, 32.2 and 18.4 months, respectively. We did not observe an effect of intrinsic subtypes on OS in this patient cohort (*p* = 0.101, Fig. 3).

Patients with Basal-like tumours showed the highest probability to pass away within two years of follow-up at 31.6% (95% CI 26,31–36,89) compared to Luminal A tumours (66.7%, 95% CI 56,51–76,89), that also corresponded to a 3.23 higher risk to die (HR 3.23, 95% CI 1.11–9.42). After adjustment to age, Nottingham grade, clinical tumour size, clinical lymph node involvement and non-Basal-like intrinsic subtypes, a higher risk to die was still detectable (HR 7.53, 95%CI 1.78–31.84). In addition, cT3/cT4 tumours were associated with an increased risk of mortality, compared to patients with cT1/cT2 tumours (HR 1.93, 95% CI 1.04–3.59). We did not observe any additional significant associations of intrinsic subtypes with OS, neither in univariate, nor in multivariate analyses (Table 4).

**Table 4:** Prognostic relevance of intrinsic subtypes in association with clinical and pathological characteristics

Factor	*n* (%)	Events	Univariate Analysis		Multivariate Analysis	
			HR (95% CI)	*p*-value	HR (95% CI)	*p*-value
age (years)						
≥ 35 (Ref)	60 (76.0%)	31				
< 35	19 (24.0%)	11	1.25 (0.62–2.51)	0.540	1.26 (0.61–2.60)	0.535
Nottingham grade						
1 or 2 (Ref)	21 (26.6%)	10				
3	58 (73.4%)	32	1.07 (0.52–2.18)	0.860	0.38 (0.13–1.05)	0.062
tumour size						
cT1 or cT2 (Ref)	35 (44.3%)	17				
cT3 or cT4	44 (55.7%)	25	**1.93 (1.04–3.59)**	**0.037**	1.73 (0.89–3.38)	0.109
lymph node status						
cN negative (Ref)	22 (27.8%)	8				
cN positive	57 (72.2%)	34	2.00 (0.92–4.34)	0.079	2.28 (0.99–5.29)	0.054
intrinsic subtype						
Luminal A (Ref)	14 (17.7%)	6				
Luminal B	31 (39.2%)	17	1.33 (0.52–3.38)	0.556	2.28 (0.67–7.76)	0.189
HER2-enriched	19 (24.1%)	10	1.68 (0.60–4.67)	0.322	3.35 (0.88–12.79)	0.077
Basal-like	15 (19.0%)	9	**3.23 (1.11–9.42)**	**0.032**	**7.53 (1.78–31.84)**	**0.006**

Bold characters denote significant HR (*p* < 0.05). The sample size for the analysis was *n* = 79. Total number of events is *n* = 42. HR, Hazard Ratio; CI, confidence interval; Ref, category taken as a reference

## Discussion

Cancer is a growing public health concern in SSA [1, 27] and the survival rates of BC are particularly low in this region [28, 29]. In recent years, GEP increasingly used in the clinical management of BC patients to gain a deeper comprehension of this heterogeneous disease. Along with histopathology and IHC for ER, PgR, Ki67 and HER2 status, GEP offer valuable information on prognosis and response to possible (neo)adjuvant treatment [9, 25, 30, 31]. However, due to high costs, GEP is often not feasible in most LMICs. However, in Brazil it has been shown that GEP using Oncotype^®^ DX could be used cost-effective for patients care [32]. To our knowledge, this is the first study that used GEP for intrinsic subtyping to evaluate its impact on OS in BC patients from rural Ethiopia without any relevant systemic treatment.

Of 113 BC patients treated only with surgery in rural Ethiopia, we described tumour biology using PAM50 gene expression assay and compared the intrinsic subtypes with clinical, histological and immunohistochemical characteristics. IHC grouping demonstrated a high prevalence of hormone receptor positive tumours of 69.0% in this BC patient cohort. Previous studies by Eng et al. [12] as well as Hercules et al. [33] found a heterogeneity of the proportions of hormone receptor positive and negative BC in different regions in SSA. Prior work from Ethiopia reported 69.0% [34] and 66.0% [35] Luminal-like subtypes, which is in line with our results. Moreover, in the US a study of BC patients found 10.3% TNBC in patients with European ancestry compared to 22.5% TNBC in non-Hispanic black patients [36], that is comparable with the proportion of TNBC (20.4%) in our cohort. Furthermore, this closely resembles findings from another Ethiopian study, which identified 23.0% [35] of tumours as TNBC. However, the proportions of TNBC found in our study slightly differ from a BC patient cohort from Mozambique (25.0% TNBC) [37] and are considerably lower than described by Ikeri et al. in a Western African cohort (42.1% TNBC) [38]. In a previous study from our group, we found a regional diversity of the distribution of IHC groups with 17.4% in Southern Africa, 22.7% in Eastern Africa and 39.4% in Western Africa [39]. Next to differences in the distribution of IHC groups in SSA population, also genetic differences have been reported [40].

In line with the IHC groups, intrinsic subtyping revealed differences in the proportions of the four subtypes compared to studies of BC patients from the US, which commonly report high numbers of Luminal A of almost half of the samples and a low amount of Basal-like subtype [13, 41]. In another study from the US among 1,319 BC specimens, Luminal A was also the most frequently identified subtype among African Americans (43.4%) [42]. This contrasts with our results, where Luminal B was the most common subtype (34.5%) and only 20.4% of Luminal A tumours could be observed. A larger cohort from Ethiopia also reported a lower proportion of Luminal A subtype compared to Western studies. [43]. In accordance with our results, a recently published South African study by Phakati et al. identified Luminal B as the most common subtype among the 377 BC patients included. Furthermore they found human immunodeficiency virus (HIV)-negativity to be associated with Luminal B subtype [44]. As the HIV status among patients in our cohort is unknown, no statement can be made on a possible correlation between intrinsic subtype and the HIV status.

Comparison of IHC groups and GEP intrinsic subtyping showed an overall good correlation, however, revealed a weakness of IHC grouping to distinguish between Luminal A-like and Luminal B-like tumours. This is also highlighted by a low Cohen’s kappa of 0.167, which is generally classified as a weak agreement. According to IHC grouping, 69.0% of tumours showed a positive hormone receptor expression and can therefore be classified as Luminal-like subtypes, and GEP identified an intrinsic Luminal subtype (Luminal A and B) in 54.9% of BC specimens, like it has been demonstrated before [19, 43]. Using GEP intrinsic subtyping half of the samples of the Luminal A-like IHC group could be allocated to Luminal B intrinsic subtype that showed a higher risk of recurrence. One reason might be due to preanalytical issues that influences the IHC results. This debility of the IHC group classification was already reported by Goldhirsch et al. [45], who suggested to employ IHC grouping as a surrogate subtype classification, while noting that GEP should be preferred to base chemotherapy decisions for patients with Luminal disease. Therefore, downstream survival analysis of our BC patient cohort was performed based only on the GEP intrinsic subtype classification. Two-year OS probability was overall 57.3% (95% CI 4.3%-69.3%) with inferior survival in women with Basal-like subtype. Patient characteristics in our cohort varied widely from those in high income countries, where mainly presentation at early stages and high survival rates are observed [46, 47]. Four in five Ethiopian women were ≤ 50 years old, more than half presented with advanced tumour size and three quarters with poorly differentiated tumours. In addition, surgery was the only treatment received by all patients of our cohort, with modified radical mastectomy (MRM) being the most common operation type. Considering mainly late presentation and lack of radiotherapy services, other surgical procedures often become infeasible [46, 47]. Kantelhardt et al. likewise identified MRM as the surgical procedure of choice in their Ethiopian cohort [4]. Tamoxifen was donated to patients with hormone receptor- positive disease from 2013 on, but due to financial hardship and poor healthcare infrastructure, only one patient adhered to endocrine therapy for more than two years [48]. Subsequently, we consider our cohort as one without any relevant (neo)adjuvant treatment received. McCormack et al. displayed that surgery as the only treatment is associated with inferior survival compared to surgery in addition to adjuvant treatment [29]. As both GEP and IHC confirm high numbers of endocrine- sensitive tumours among women of our East African cohort, access to Tamoxifen and proper long-term implementation of endocrine treatment is essential to improve survival rates. We suggest as demonstrated by Getachew et al. the deployment of trained Breast Nurses to enhance adherence to adjuvant endocrine treatment [49]. Furthermore, as PAM50 subtype distribution showed a large proportion of Luminal B, HER2-enriched and Basal-like tumours additional implementation of chemotherapy would be of advantage to many BC patients in the district of Aira.

Due to late presentation of patients at advanced tumour stages and lack of neoadjuvant or adjuvant therapy regimes this very poor clinical outcome was found. Similar crude two-year survival rates of approximately 60% in stage III BC patients were reported in the African Breast Cancer – Disparities In Outcome (ABC-DO) study [29] Patients of our cohort presenting with cT3/cT4 tumours showed inferior survival compared to more favourable cT1/cT2 tumours. Our results confirm the widely recognized association between large tumour size and poor prognosis [50]. However, in our cohort, patients with Basal-like tumours had worse outcome compared to all other subtypes (31.6% vs. 62.9% survial probability after 24 months) and demonstrated to be a stronger prognostic factor when compared with tumour size. Risk of dying at two years was lowest for Luminal A tumours, with a mean subtype-specific survival time of 43.6 months vs. 18.4 months in patients with Basal-like subtype. These findings are in line with other studies in the US [10, 51], even though OS rates are considerably higher than in our cohort. Phakathi et al. likewise reported differences in GEP subtype-specific survival among HIV-negative patients in their South African cohort [44]. Patients with luminal BC subtypes showed superior survival compared to non-luminal BC subtypes [44]. When we used log-rank test to investigate an effect of intrinsic subtype on survival probability, we did not find any evidence when testing for all four subtypes. One reason for that could be the lack of (neo)adjuvant treatment in our cohort. In comparison, the HIV-negative patients investigated by Phakati and colleagues received systemic treatment with chemotherapy and endocrine treatment but no anti-HER2 therapy [44, 52, 53]. This presumably resulted in superior survival of not only Luminal A but also Luminal B (HR 2.39) BC subtype compared to lower OS probability of patients with HER2-enriched (HR 6.09) and Basal-like subtype (HR 5.47) [44, 53]. However, paucity of data regarding GEP subtype-specific survival in SSA and differences in treatment make the comparison arduous.

### Strengths and limitations

To our knowledge, this is the first study that investigated the impact of PAM50 intrinsic BC subtypes on survival of a patient cohort without any (neo)adjuvant treatment of women with BC living in a rural East African area. Through active follow-up including home visits and phone calls, precise information on the patients’ survival status was available. However, follow-up could not be obtained from some patients because they could not be contacted by telephone, or no relatives could be found who could provide relevant data. An additional limitation of this study is the small sample size with possible selection bias as this was not a population-based but a single-center hospital-based cohort. However, the proportions of intrinsic subtypes are comparable with other studies reported in the region. Furthermore, due to the low number of previous research data available, contextualisation and interpretation of our findings is still difficult. Further research using GEP for BC intrinsic subtype classification is needed to gain a deeper understanding of geographic and inter-ethnic differences in tumour biology and survival of BC patients in East Africa.

## Conclusion

In our rural patient cohort, nearly half of the female BC patients treated only with surgery died within two years after receiving their diagnosis. An unfavourable prognosis was observed for patients that presented with large tumour size (> 5 cm) and for those that were classified as Basal-like intrinsic subtype compared to patients with non-Basal-like intrinsic subtypes. Both, IHC grouping and intrinsic subtypes showed a large share of endocrine responsive tumours. However, Luminal B intrinsic subtype was the most common intrinsic subtype. This indicates the need to promote both the implementation of endocrine therapy and offer access to chemotherapy in rural Ethiopia in order to improve BC patients’ outcomes. Moreover, a local implementation of BC IHC grouping should be pursued in the routine diagnosis for therapy recommendations.

## Electronic supplementary material

Below is the link to the electronic supplementary material.

Supplementary Material 1

Supplementary Material 2

## Data Availability

All data generated in this study are available upon request to the corresponding author.

## Abbreviations

ASC-CAP: American Society of Clinical Oncology and College of American Pathologists
BC: Breast cancer
CI: Confidence interval
ER: Estrogen receptor
ENCR: European network of cancer registries
FFPE: Formalin-fixed, paraffin-embedded
GEP: Gene expression profiling
HER2: Human Epidermal Growth Factor 2
HIV: Human immunodeficiency virus
HR: Hazard ratio
IHC: Immunohistochemistry
LMIC: Low- and middle-income countries
MRM: Modified radical mastectomy
NST: No special type
OS: Overall survival
PAM50: Prediction Analysis of Microarray 50
PgR: Progesterone receptor, SSA, sub-Saharan Africa
TNBC: Triple negative breast cancer
WHO: World Health Organization
