# Supplementary material for: PAM50 breast cancer subtypes and survival of patients in rural Ethiopia without adjuvant treatment: a prospective observational study
Source: BMC Cancer. 2024 Sep 10;24:1127. doi: 10.1186/s12885-024-12867-6 (PMC11385137; doi:10.1186/s12885-024-12867-6)
Supplement: Supplementary file 1 — Supplementary Material 1 [file 12885_2024_12867_MOESM1_ESM.docx]

Supplementary Table S1: Overview of antibodies applied

| **Antibody** | **clone** | **supplier** |
| --- | --- | --- |
| Estrogen receptor (ER) | 1D5 | Thermo Scientific, MA, USA |
| Human Epidermal Growth Factor 2 (Her2) | DG44 | DAKO, CA, USA |
| Ki67 | SP6 | Thermo Scientific, MA, USA |
| Progesterone receptor (PgR) | PgR 636 | DAKO, CA, USA |
