# Supplementary material for: PAM50 breast cancer subtypes and survival of patients in rural Ethiopia without adjuvant treatment: a prospective observational study
Source: BMC Cancer. 2024 Sep 10;24:1127. doi: 10.1186/s12885-024-12867-6 (PMC11385137; doi:10.1186/s12885-024-12867-6)
Supplement: Supplementary file 2 — Supplementary Material 2 [file 12885_2024_12867_MOESM2_ESM.docx]

Supplementary Table S2: Comparison of patients with and without follow up data

| Characteristics | **patients with survival data** | **patients without survival data** | **p-value**  $\mathbf{(}$**Fisher’s exact test)** |
| --- | --- | --- | --- |
| n | 79 | 34 |  |
| age (years) |  |  | 0.198 |
| <35 | 19 | 11 |  |
| 35-50 | 45 | 16 |  |
| >50 | 15 | 2 |  |
| hormone receptor status |  |  | 0.515 |
| negative (35) | 23 | 12 |  |
| positive (78) | 56 | 22 |  |
| intrinsic subtype |  |  | 0.273 |
| Luminal A | 14 | 9 |  |
| Luminal B | 31 | 8 |  |
| HER2-enriched | 19 | 7 |  |
| Basal-like | 15 | 10 |  |
